# Consumption of sugar sweetened beverage is associated with incidence of metabolic syndrome in Tehranian children and adolescents

**DOI:** 10.1186/s12986-015-0021-6

**Published:** 2015-07-30

**Authors:** Parvin Mirmiran, Emad Yuzbashian, Golaleh Asghari, Somayeh Hosseinpour-Niazi, Fereidoun Azizi

**Affiliations:** Nutrition and Endocrine Research Center, Research Institute for Endocrine Sciences, Shahid Beheshti University of Medical Sciences, Tehran, Iran; Endocrine Research Center, Research Institute for Endocrine Sciences, Shahid Beheshti University of Medical Sciences, Tehran, Iran

**Keywords:** Metabolic syndrome, Children, Adolescents, Sugar sweetened carbonated soft drink, Fruit juice drink, Sugar sweetened beverage

## Abstract

**Background:**

Intakes of high sugar-sweetened beverages (SSBs) in adults can escalate risk of metabolic syndrome (MetS); however, data of longitudinal studies in children and adolescents are lacking. In this study we assessed consumption of SSBs in relation to incidence of MetS among children and adolescents during a 3.6 year follow-up.

**Methods:**

This study was a population-based longitudinal study, in which 424 subjects, aged 6–18 years, from the Tehran Lipid and Glucose Study with complete data on dietary intake, blood pressure, anthropometry, and biochemical indices were followed for 3.6 years. Dietary intake was collected using a valid and reliable food frequency questionnaire. MetS was defined according to the Cook criteria. Sugar sweetened beverages included all kinds of sugar sweetened carbonated soft drinks (SSSDs) and fruit juice drinks.

**Results:**

Average daily intakes of SSSD and fruit juice drinks were 38.5 ± 75.0 and 32.3 ± 60.1 g, respectively. After adjustment for confounders, compared to the first quartile, the odds ratio of incident MetS in the highest quartile of SSB and SSSD was 3.20 (95 % CI: 1.06–9.90) and 3.01 (95 % CI: 1.17–7.74), respectively. Regarding incidence of MetS components, compared with the lowest quartile, the highest quartile of SSSDs showed odds ratios of 2.49 (95 % CI: 1.00–6.53) for abdominal obesity and 2.79 (95 % CI: 1.02–7.64) for hypertension. No significant association was found between consumption of fruit juice drink and SSSD with other components of MetS.

**Conclusions:**

Children and adolescents with high intakes of carbonated beverages could be at increased risk of MetS, abdominal obesity, and hypertension.

## Introduction

The rising prevalence of metabolic syndrome (MetS) during childhood and adolescence affectes over 3.3 % of the adolescent population worldwide [1], and may be predictive of adult MetS, type 2 diabetes, and cardiovascular disease [2, 3]. Although some studies have examined genetic predispositions, findings show that only 10 % of MetS cases can be explained by genetics [4]. Alternatively, many environmental factors have been identified [5], e.g. food intakes play an important role in the pathogenesis of MetS through hepatic insulin resistance and/or increasing reactive oxygen species formation [6].

Nowadays the consumption of sugar sweetened beverages (SSBs) has increased drastically particularly in children and adolescents [7–9]. Higher SSB intakes in adults may enhance risk of weight gain and of developing obesity and obesity-related disorders, such as MetS, type 2 diabetes, coronary heart disease, and stroke [10, 11]. However, according to a recent evidence mapping study by Althuis et al. [12], longitudinal studies in children and adolescents are lacking and limited to one cross-sectional investigation, which indicated that SSBs have been linked to increased risk of MetS, hypertension, and abdominal obesity [13].

In the current study, we evaluated the association between SSB consumption with incident MetS and its components 3.6 years later among children and adolescents in Iran.

## Materials and method

Tehran Lipid and Glucose Study (TLGS) is a prospective community-based study being conducted to determine the risk factors of and prevent non-communicable diseases by implementing and promoting healthy lifestyles [14].

Baseline data were collected from 15005 participants, aged 3 years and over, of residents District No. 13 of Tehran under coverage of three medical health centers. The participants were followed up every 3 years to update their data on demographic, lifestyle, biochemical, clinical, and dietary measurements; the baseline survey was a cross-sectional study conducted from 1999 to 2001, and surveys 2 (2002–2005), 3 (2006–2008), and 4 (2009–2011) were prospective follow-up surveys.

In the third survey of the TLGS (2006–2008), from among 12523 examined participants, 3462 were randomly selected for dietary assessment; of these, 621 individuals were aged 6–18 y who included in the current study. Participants who had incomplete dietary intakes or had missing measures of MetS components (*n* = 29) were excluded. Additionally, participants with reported energy intakes (kcal/day) to energy requirements ratios beyond ±3SD range, were also excluded (*n* = 6) because they fell into the over-or under-reporting categories [15]. Participants were followed until survey 4 (2009–2011), an average of 3.6 years (follow-up rate: 86 %). Depending on the outcome variable under analysis, we excluded persons whose data at survey 3 (2006–2008) indicated prevalent MetS (*n* = 69), hypertension (*n* = 53), high triglycerides (TGs) (*n* = 168), low high density lipoprotein cholesterol (HDL-C) (*n* = 242), high fasting plasma glucose (FPG) (*n* = 12), or abdominal obesity (*n* = 145); some individuals fell into more than one exclusion category. The final sample sizes in fully adjusted models varied by outcomes as follows: MetS (*n* = 424), abdominal obesity (*n* = 327), high triglycerides (TGs) (*n* = 347), hypertension (*n* = 439), low HDL-C (*n* = 290), and high fasting plasma glucose (*n* = 476).

The design of this study was approved by the institutional ethics committee of the Research Institute for Endocrine Sciences, Shahid Beheshti University of Medical Sciences, and written informed consent was obtained from participants’ parents.

### Dietary intake

A valid and reliable 168-item semi-quantitative food frequency questionnaire (FFQ) [16–18] was used by trained dietitians, during face-to face interviews, to evaluate the usual dietary intakes of participants. Participants were asked to report their consumption frequency of food items during the previous year on a daily, weekly, or monthly basis. Mothers were asked about the type and quantity of meals and snacks when children were unable to recall. Portion sizes of consumed foods were specified according to the US Department of Agriculture (USDA) standard portion sizes or household measures (e.g. beans, 1 tablespoon; chicken meat, 1 leg or wing; rice, 1 large or small plate) [19]. Obtained data were converted to mean daily intake in grams or milliliters. This FFQ included questions on the frequency of consumption and usual portion size of all kinds of sugar sweetened carbonated soft drinks (SSSDs) and fruit juice drinks (both 100 % fruit juice and sugar sweetened synthetic juice drinks that are not 100 % juice). According to Althuis et al. [12], these items were combined to estimate the daily intake of SSBs (ml/d). SSB intakes were divided by using quartile cutoffs, with the assumption that one serving was equivalent to 1 cup (250 mL).

To study the reproducibility of the FFQ, 132 subjects (61 men and 71 women) twice completed a 168-item FFQ (FFQ1, FFQ2), with a 14-month interval between FFQ1 and FFQ2, and to assess the validity, 12 dietary recalls (DRs) were collected (1 each month) over the 1-year interval. The age-and energy-adjusted and deattenuated Spearman correlation coefficients used to assess validity of the FFQ for soft drinks were 0.54 and 0.37 in men and women, respectively. The age-and energy-adjusted intraclass correlation coefficients, which reflect the reproducibility of food groups in the FFQ, were 0.61 and 0.65 in men and in women, respectively [17].

### Measurements

Information about physical activity was collected using the modifiable activity questionnaire (MAQ) to calculate metabolic equivalent task (MET) minutes per week [20]. High reliability (98 %) and moderate validity (47 %) were ascertained for the MAQ translated into Persian [21]. Levels of physical activity were categorized as light (MET <600 min/wk), moderate (MET 600–1,499 min/wk), and vigorous (MET ≥1,500 min/wk).

Weight was measured, while participants were minimally clothed without shoes, using digital scales (Seca 707, Seca Corp., Hanover, MD; range 0.1–150 kg) and recorded to the nearest 100 g. Height was measured in a standing position without shoes, using a stadiometer, with shoulders in normal alignment. Body mass index (BMI) was calculated as weight (kg) divided by the square of height (m^2^). Waist circumference (WC) was measured at the umbilicus, using a measuring tape, without pressure to body surfaces, and was recorded to the nearest 0.5 cm.

Blood pressure was measured twice, after participants were seated for 15 min, using a standard mercury sphygmomanometer; there was at least a 30-s interval between these two separate measurements, and the mean of two measurements was considered the patient’s blood pressure.

Blood samples, at baseline and follow up, were drawn between 7:00 and 9:00 AM from all study participants after 12–14 h overnight fasting; all blood analyses were done at the TLGS research laboratory on the day of blood collection. FPG was measured by the enzymatic colorimetric method using glucose oxidase. Serum HDL-C was measured after precipitation of the apolipoprotein B-containing lipoproteins with phosphotungstic acid and serum TGs were assayed using an enzymatic colorimetric method with glycerol phosphate oxidase. These analyses were performed using commercial kits (Pars Azmoon Inc., Tehran, Iran) and a Selectra 2 auto analyzer (Vital Scientific, Spankeren, The Netherlands). Inter-and intra-assay coefficients of variations at baseline were both 2.2 % for FPG, 2 and 0.5 % for HDL-C and 1.6 and 0.6 % for TGs, respectively.

### Definitions

In children and adolescents, MetS was defined according to definition proposed by Cook et al. as 3 or more of the following [22]: Fasting TGs ≥110 mg/dl; HDL-C < 40 mg/dl; WC ≥ 90th percentile for age and sex, according to national reference curves [23]; systolic blood pressure and diastolic blood pressure ≥90th percentile for sex, age and height, from the National Heart, Lung, and Blood Institute’s recommended cut-off points [24], and FPG ≥ 100 mg/dl, according to the recommendations of American Diabetes Association [25]. In subjects aged > 18 years after follow-up, MetS was defined according to the joint interim statement [26] as the presence of any 3 of 5 risk factors of the following: 1) Abdominal obesity as WC ≥91 and men ≥89 cm for women and men, respectively, according to Iranians cut-off points [27]; 2) FPG ≥100 mg/dl or drug treatment; 3) fasting TGs ≥150 mg/dl or drug treatment; 4) fasting HDL-C <50 mg/dl for women and <40 mg/dl for men or drug treatment, and 5) hypertension was defined as systolic blood pressure ≥130 mmHg, diastolic blood pressure ≥85 mmHg, or antihypertensive drug treatment.

### Statistical analysis

All analyses were conducted using the Statistical Package for Social Sciences (version 15.0; SPSS Inc, Chicago IL). Baseline characteristics of subjects were expressed as mean ± SD or median (interquartile 25–75) for continuous variables and percentages for categorical variables. To investigate the trend of continuous and categorical variables according to quartiles of SSB, linear regression and chi-square test were used, respectively. There were no significant differences in baseline anthropometric and biochemical assessments between subjects who provided follow-up assessments and those lost to follow-up (data not shown).

Intakes of SSBs (<19.2, 19.2-43.8, 43.9-91.5, and >91.6 ml/day), SSSDs (<7.6, 7.6–15.3, 15.3–40.0, and >40.0 ml/day), and fruit juice drinks (<4.1, 4.1–13.5, 13.5–34.9, and 34.9 ml/day) were categorized into the quartile cut off points. To estimate the risk of 3.6-year incident outcomes, separate multiple regression models were used for abdominal obesity, high FPG, high TGs, low HDL-C, hypertension, and MetS with quartiles of SSB, SSSD, and fruit juice drink consumption; odds ratios (OR) and 95 % confidence interval (CI) were reported. The initial model was adjusted for age, sex, total energy intake, physical activity, and family history of diabetes. Further adjustment for dietary fiber, tea and coffee, red and processed meat, fruits, and vegetables was made and the final model was additionally adjusted for BMI.

## Results

The mean ± SD age of study population (68 % girls) was 13.6 ± 3.7 years; the mean daily SSB intake was 98 ml in boys and 70 ml in girls. After a 3.6 year follow up, cumulative incidence of MetS was 11 %.

The descriptive characteristics of the study population according to quartile of SSB consumption are shown in Table 1. Participants with higher SSB consumption were likely to have higher systolic blood pressure, FPG, and BMI; in addition, subjects with higher SSB consumption had higher intakes of daily energy, saturated fatty acids, trans-fatty acids, glucose, fructose, sucrose, simple sugars, higher glycemic index, and lower intakes of fruits and whole grains.

**Table 1:** Baseline characteristics of children and adolescents according to quartiles of sugar sweetened beverage consumption: Tehran Lipid and Glucose Study

	Quartiles				*P* for trend^1^
	Q1 (*n* = 106)	Q2 (*n* = 106)	Q3 (*n* = 106)	Q4 (*n* = 106)	
Median intake (ml/d)	9.3	32.0	58.6	142.2	
Age (years)	13.8 ± 3.7	12.9 ± 3.7	13.8 ± 3.7	13.7 ± 3.6	0.737
Girls (%)	72	43	62	50	0.092
Systolic blood pressure (mmHg)	96.8 ± 12.2	97.7 ± 10.7	97.1 ± 11.7	100.2 ± 12.0	0.021
Diastolic blood pressure (mmHg)	64.3 ± 9.4	63.9 ± 6.9	65.7 ± 9.1	64.7 ± 10.2	0.520
Triglycerides (mg/dl)	83.0 (62.7–103.2)	77.0 (63.0–94.0)	84.0 (64.0–105.2)	81.5 (63.0–112.5)	0.216
Fasting plasma glucose (mg/dl)	83.9 ± 5.9	84.4 ± 5.9	85.5 ± 5.8	86.0 ± 6.5	0.037
High density lipoprotein (mg/dl)	45.8 ± 11.9	46.3 ± 11.11	47.3 ± 9.6	43.8 ± 9.4.2	0.269
Waist circumstance (cm)	69.7 ± 11.1	69.7 ± 10.4	67.8 ± 10.6	68.1 ± 9.5	0.231
Body mass index (kg/m^2^)	18.7 ± 4.3	19.2 ± 3.8	19.7 ± 3.6	20.1 ± 3.8	<0.001
*Daily dietary intakes*					
Total energy (kcal)	2116 ± 962	2364 ± 1017	2440 ± 1020	3317 ± 1670	<0.001
Dietary fiber/1000 kcal (g)	14.5 ± 6.4	14.4 ± 6.6	14.6 ± 5.5	14.2 ± 4.6	0.866
Total fat (% energy)	32.4 ± 8.5	31.9 ± 6.3	32.3 ± 7.2	31.6 ± 5.5	0.785
Trans-fatty acids (g)	4.17 ± 3.88	4.23 ± 2.83	5.26 ± 5.77	6.6 ± 4.6	<0.001
Saturated fatty acids (g)	25.1 ± 12.7	28.1 ± 13.2	27.6 ± 16.4	41.9 ± 23.2	<0.001
Protein (% energy)	13.0 ± 2.4	13.8 ± 3.4	12.8 ± 1.9	12.9 ± 1.9	0.230
Carbohydrate (% energy)	56.2 ± 8.7	56.8 ± 7.4	57.1 ± 7.1	57.5 ± 5.9	0.098
Glucose (g)	11.7 ± 7.8	14.7 ± 7.5	15.2 ± 7.2	25.2 ± 15.2	<0.001
Fructose (g)	13.2 ± 8.8	16.5 ± 8.2	18.2 ± 8.7	23.9 ± 7.9	<0.001
Sucrose (g)	26.8 ± 21.2	30.6 ± 20.3	36.5 ± 60.4	41.1 ± 28.4	<0.001
Total simple sugar (g)	111.4 ± 81.8	118.7 ± 56.0	124.3 ± 81.9	142.9 ± 98.0	<0.001
Total simple sugar (% energy)	20.8 ± 7.7	20.3 ± 5.2	20.2 ± 5.76	21.6 ± 5.7	0.422
Glycemic index	61.1 ± 35.8	78.4 ± 38.2	81.5 ± 45.11	102.1 ± 74.5	<0.001
Fruit (g/1000 kcal)	183.4 ± 118	173.5 ± 113.6	160.5 ± 94.4	130.4 ± 100.1	<0.001
Vegetable (g/1000 kcal)	105.7 ± 66.2	94.8 ± 55.8	97.4 ± 53.5	94.5 ± 46.2	0.200
Low fat dairy (g/1000 kcal)	117.9 ± 90.3	113.9 ± 89.7	109.1 ± 75.3	121.5 ± 91.9	0.875
Meat and processed meat (g/1000 kcal)	11.4 ± 11.9	12.9 ± 9.4	12.5 ± 10.1	14.9 ± 11.2	0.076
Legumes (g/1000 kcal)	7.0 ± 8.9	6.9 ± 7.6	7.3 ± 9.1	5.9 ± 7.1	0.362
Whole grains (g/1000 kcal)	38.7 ± 40.9	30.9 ± 31.6	27.0 ± 25.0	26.5 ± 26.6	0.003

Data represented as mean ± SD or median (IQ 25–75) for continuous variables and percentage for categorically distributed variables

^1^Based on linear regression models for continuous variables and chi-square for categorical variables

Sugar sweetened beverage intake was inversely related to MetS and its components after adjustment for potential confounders (Table 2). Further adjustment for dietary factors did not have a major impact on the results. In this model, subjects in the highest quartile of SSB consumption had increased risk of incident MetS (OR: 3.16; 95 % CI: 1.02–9.78, *P* for trend = 0.007) and abdominal obesity (OR: 2.97; 95 % CI: 1.23–7.19, *P* for trend = 0.017). Adjustment for BMI slightly strengthened the association; adjusted multivariable ORs of MetS, abdominal obesity, and hypertension were 3.50, 3.66, and 2.90 (all *P* for trends < 0.05).

**Table 2:** Multivariable-adjusted ORs (95 % CIs) of incident MetS and its components by quartiles of sugar sweetened beverage consumption after 3.6 years of follow up among children and adolescents

	Quartiles				P for trend^1^
	Q1	Q2	Q3	Q4	
Median intake (ml/d)	9.3	32.0	58.6	142.2	
MetS					
Model 1	1.00	1.06 (0.31–3.57)	2.84 (0.97–8.37)	3.00 (0.99–9.05)	0.009
Model 2	1.00	1.02 (0.30–3.48)	2.94 (0.99–8.74)	3.16 (1.02–9.78)	0.007
Model 3	1.00	1.11 (0.32–3.82)	2.96 (0.99–8.80)	3.50 (1.12–10.92)	0.005
Low HDL-C					
Model 1	1.00	0.72 (0.24–2.16)	0.96 (0.33–2.82)	0.55 (0.17–1.81)	0.434
Model 2	1.00	0.61 (0.19–1.89)	0.93 (0.31–2.78)	0.42 (0.11–1.55)	0.320
Model 3	1.00	0.65 (0.21–2.07)	0.97 (0.32–2.93)	0.45 (0.12–1.66)	0.386
Abdominal obesity					
Model 1	1.00	1.53 (0.63–3.71)	1.65 (0.61–3.94)	2.94 (1.27–6.82)	0.012
Model 2	1.00	1.58 (0.65–3.86)	1.70 (0.70–4.09)	2.97 (1.23–7.19)	0.017
Model 3	1.00	2.16 (0.82–5.68)	1.86 (0.71–4.84)	3.66 (1.40–9.59)	0.016
Hypertension					
Model 1	1.00	1.46 (0.45–4.77)	2.66 (0.89–7.96)	2.41 (0.79–7.73)	0.070
Model 2	1.00	1.47 (0.45–4.82)	2.68 (0.89–8.11)	2.45 (0.78–7.70)	0.072
Model 3	1.00	1.73 (0.52–5.74)	3.02 (0.98–9.25)	2.90 (0.91–9.26)	0.043
High FPG					
Model 1	1.00	1.22 (0.56–3.22)	1.90 (0.76–4.72)	2.07 (0.79–5.39)	0.079
Model 2	1.00	1.17 (0.44–3.08)	1.83 (0.73–4.58)	1.90 (0.71–5.09)	0.109
Model 3	1.00	1.21 (0.48–3.21)	1.87 (0.75–4.68)	1.95 (0.73–5.22)	0.108
High TGs					
Model 1	1.00	0.76 (0.24–2.38)	1.57 (0.57–4.33)	1.70 (0.58–4.99)	0.156
Model 2	1.00	0.74 (0.23–2.33)	1.53 (0.55–4.29)	1.66 (0.55–5.05)	0.173
Model 3	1.00	0.82 (0.26–2.61)	1.62 (0.57–4.58)	1.80 (0.59–5.25)	0.148

*MetS* metabolic syndrome, *HDL-C* high density lipoprotein-cholesterol, *FPG* fasting plasma glucose, *TGs* triglycerides

Model1: Adjusted for baseline age, sex, total energy intake, physical activity, and family history of diabetes

Model 2: Additionally adjusted for dietary fiber, tea and coffee, red and processed meat, fruit, and vegetable

Model 3: Additionally adjusted for body mass index

^1^Based on logistic regression model using median intake of sugar sweetened beverages in each quartile as a continuous variable

Higher intakes of SSSD was associated with significant increase in the risk of incident MetS (OR: 3.58, 95 % CI: 1.09-11.78, *P* for trend = 0.013), abdominal obesity (OR: 3.78, 95 % CI: 1.08–13.27, *P* for trend = 0.043), and hypertension (OR: 2.74, 95 % CI: 1.05–7.19, *P* for trend = 0.018) in the fully adjusted model. Associations for fruit juice drink intake were not statistically significant (Table 3).

**Table 3:** Multivariable-adjusted ORs (95 % CIs) of incident MetS and its components by quartiles of sugar sweetened carbonated soft drink and fruit juice drink intakes after 3.6 years of follow up among children and adolescents

	Quartiles				*P* for trend^1^
	Q1	Q2	Q3	Q4	
Sugar sweetened carbonated soft drink (ml/d)	1.12	9.33	33.06	100.00	
MetS					
Model 1	1.00	1.06 (0.31–3.57)	2.29 (0.76–6.97)	3.33 (1.04–10.73)	0.014
Model 2	1.00	1.02 (0.30–3.47)	2.39 (0.78–7.31)	3.45 (1.06–11.18)	0.012
Model 3	1.00	1.11 (0.32–3.82)	2.44 (0.79–7.49)	3.58 (1.09–11.78)	0.013
Low HDL-C					
Model 1	1.00	0.38 (0.12–1.30)	0.77 (0.31–1.93)	1.00 (0.33–2.98)	0.982
Model 2	1.00	0.37 (0.11–1.27)	0.71 (0.28–1.84)	1.15 (0.35–3.79)	0.950
Model 3	1.00	0.37 (0.11–1.28)	0.69 (0.26–1.80)	1.15 (0.35–3.81)	0.989
Abdominal obesity					
Model 1	1.00	1.73 (0.72–4.16)	1.99 (0.83–4.77)	2.76 (0.92–8.28)	0.064
Model 2	1.00	1.85 (0.76–4.49)	2.23 (0.91–5.43)	3.39 (1.08–10.70)	0.034
Model 3	1.00	1.57 (0.60–4.06)	2.03 (0.78–5.28)	3.78 (1.08–13.27)	0.043
Hypertension					
Model 1	1.00	0.77 (0.25–2.36)	1.29 (0.46–3.57)	2.64 (1.03–6.80)	0.022
Model 2	1.00	0.80 (0.26–2.44)	1.36 (0.48–3.84)	2.84 (1.04–7.19)	0.021
Model 3	1.00	0.79 (0.26–2.44)	1.37 (0.49–3.84)	2.74 (1.05–7.19)	0.018
High FPG					
Model 1	1.00	0.58 (0.21–1.60)	1.97 (0.86–4.53)	1.12 (0.41–3.10)	0.243
Model 2	1.00	0.56 (0.20–1.56)	1.95 (0.84–4.54)	1.11 (0.40–3.11)	0.255
Model 3	1.00	0.55 (0.20–1.54)	1.93 (0.83–4.50)	1.12 (0.40–3.12)	0.251
High TGs					
Model 1	1.00	1.04 (0.35–3.11)	2.01 (0.71–5.68)	1.89 (0.65–5.60)	0.137
Model 2	1.00	1.03 (0.34–3.12)	2.04 (0.71–5.89)	1.97 (0.46–6.02)	0.124
Model 3	1.00	0.83 (0.27–2.58)	1.91 (0.66–5.50)	1.75 (0.57–5.37)	0.148
Fruit juice drink (ml/d)	1.31	8.29	20.40	67.14	
MetS					
Model 1	1.00	1.17 (0.46–2.96)	0.62 (0.22–1.79)	1.18 (0.46–3.06)	0.993
Model 2	1.00	1.10 (0.42–2.82)	0.60 (0.20–1.78)	1.17 (0.42–3.16)	0.984
Model 3	1.00	1.13 (0.43–2.91)	0.64 (0.21–1.92)	1.19 (0.44–3.21)	0.947
Low HDL-C					
Model 1	1.00	0.76 (0.27–2.14)	0.76 (0.29–2.01)	0.49 (0.16–1.49)	0.235
Model 2	1.00	0.68 (0.23–1.99)	0.70 (0.26–1.86)	0.43 (0.14–1.36)	0.171
Model 3	1.00	0.69 (0.23–2.02)	0.72 (0.27–1.92)	0.44 (0.14–1.39)	0.181
Abdominal obesity					
Model 1	1.00	1.54 (0.71–3.35)	0.75 (0.31–1.80)	1.32 (0.59–2.95)	0.899
Model 2	1.00	1.53 (0.69–3.78)	0.72 (0.29–1.77)	1.15 (0.48–2.77)	0.854
Model 3	1.00	2.31 (0.95–5.61)	0.88 (0.33–2.35)	1.26 (0.48–3.34)	0.865
Hypertension					
Model 1	1.00	1.72 (0.63–4.72)	1.48 (0.53–4.14)	1.10 (0.38–3.21)	0.990
Model 2	1.00	1.86 (0.67–5.19)	1.65 (0.57–4.77)	1.18 (0.39–3.58)	0.878
Model 3	1.00	2.00 (0.71–5.66)	1.91 (0.65–5.60)	1.28 (0.42–3.94)	0.748
High FPG					
Model 1	1.00	1.17 (0.46–2.97)	0.62 (0.22–1.79)	1.18 (0.46–3.06)	0.993
Model 2	1.00	1.10 (0.43–2.82)	0.60 (0.20–1.78)	1.17 (0.44–3.16)	0.984
Model 3	1.00	1.13 (0.44–2.91)	0.64 (0.21–1.92)	1.19 (0.44–3.21)	0.947
High TGs					
Model 1	1.00	0.62 (0.23–1.63)	0.49 (0.18–1.31)	0.79 (0.30–2.07)	0.464
Model 2	1.00	0.60 (0.22–1.60)	0.43 (0.15–1.20)	0.77 (0.28–2.11)	0.419
Model 3	1.00	0.64 (0.24–1.73)	0.47 (0.16–1.34)	0.84 (0.30–2.33)	0.544

*MetS* metabolic syndrome, *HDL-C* high density lipoprotein-cholesterol, *FPG* fasting plasma glucose, *TGs* triglycerides

Model1: Adjusted for baseline age, sex, total energy intake, physical activity, and family history of diabetes

Model 2: Additionally adjusted for dietary fiber, tea and coffee, red and processed meat, fruit, and vegetable

Model 3: Additionally adjusted for body mass index

^1^Based on logistic regression model using median intake of sugar sweetened beverages in each quartile as a continuous variable

## Discussion

In the present study, we provided evidence that children and adolescents who consumed SSB over 90 ml/day (one third of a serving) had almost a 3-fold increased risk of incident MetS, abdominal obesity, and hypertension independent of confounders, particularly BMI. Similarly, higher SSSD consumption was directly associated with incidence of MetS, abdominal obesity, and hypertension.

Findings of the current study support those of prospective ones conducted on SSBs and MetS in adults [28, 29], which report that adults with higher SSB consumption had a 0.5–2 fold increased risk of incident MetS. To our knowledge, the present study is the first to examine the association of SSB, SSSD, and fruit juice drink consumption with MetS in longitudinal analysis in children and adolescents. Our findings are consistent with previously published cross-sectional reports in which high SSBs consumption was associated with prevalent MetS and higher insulin resistance in children and adolescents [13, 30, 31]. Adolescents, aged 12 to 16 years, with higher consumption of SSB had a 5.1-fold increased prevalence of MetS [13]. Also each additional SSB serving intake was associated with a 5 % increment of insulin resistance among 12–19 year-old American adolescents [31]. In addition, higher SSB consumption in children, aged 8–10 years, was associated with a 0.1-unit higher HOMA-IR [30]. Our prospective findings confirmed the proposed causal link in cross-sectional studies between SSB consumption, insulin resistance, and MetS.

Sugar sweetened carbonated soft drinks are among the components of SSB and in the current study, had a positive association with incident MetS, abdominal obesity, and hypertension. Similarly, these beverages are associated with greater odds of unfavorable changes in cardio-metabolic risk factors, independent of body weight [31, 32]. The Western Australian Cohort (Raine) Study showed that SSB consumption of over 1.3 servings/d, was associated with a 3.2-fold increment of overall cardio-metabolic risk, and had unfavorable effects on changes of waist circumference, TGs, and HDL-C, independent of BMI after a 2-year follow-up [32]. Our results are also consistent with a cross-sectional study of 12–19 year-old American adolescents, who consumed on average 7.4 servings of SSBs daily, indicating that each additional SSB serving intake was associated with increments of systolic blood pressure and waist circumference, and with decreased HDL-C, after adjusting for age, sex, race, menarche, and energy intake, but not for BMI [31]. Interestingly, our study found an approximately 3.6-fold greater risk of MetS following consumption of a one third of serving of SSSDs comparable to results of studies with similar cardio-metabolic risks [32] but with much higher consumption, highlighting the magnitude of this association in the current study.

Dietary behavior, socio-economic factors, and physiological effects explain the unfavorable effects of higher SSB consumption on metabolic abnormalities [29]; subjects with increased SSB consumption may show properties of unhealthy dietary pattern [33]. In the current study, individuals with higher SSB consumption had higher intakes of total energy, trans and saturated fatty acids, fructose, simple sugars, and glycemic load. Lower socioeconomic status and increased hours of watching TV were associated with increased SSB intake; children from low socioeconomic status areas consumed sugar sweetened soft drinks approximately twice as common [34]. In the current investigation, 35 % parents of children studied had education levels < 12 years, which is an important point to consider in interpretation of findings. Several studies highlight the potential for parents to influence SSB intakes of children and adolescents, possibly as they are the main purchasers of food and beverages consumed; those who have parents or caregivers with high education status consume lower amounts of SSBs compared to those who have parents with lower levels of education [32]. Because SSBs are in liquid form, they induce lower satiety and higher energy intake, compared to solid form of foods [10]. Fruit juice drinks in Iran include both natural and synthetic fruit juices with added sugars. Compared to SSSDs and fruit juice drinks, other sugar sweetened beverages such as beverages made with powdered flavoring, non-juice fruit-flavored drinks, sports drinks, artificial non-caloric carbonated soft drinks, or sweetened coffee drinks are far less frequently consumed in Iran, they were not considered in our analysis. Higher SSB consumption has indirect and direct associations with insulin resistance and MetS; the direct association is due to the glycemic effects of the large amounts of absorbed sugars or metabolic effects of fructose and the indirect association because of weight gain. Evidence indicates that higher SSB consumption is concomitant with excess body weight gain, which may lead to unhealthy metabolic homeostasis [35, 36]. In the current study, BMI rose according to the quartiles of SSB consumption; however, the associations between incident MetS and higher SSB intakes remained significant even after controlling for BMI, indicating that SSB may affect insulin resistance via pathways that do not include excess weight. The physiological effect of SSB consumption on metabolic abnormalities may be explained by high dietary glycemic load leading to a postprandial rise in blood glucose and insulin concentrations, which over time may lead to hyperinsulinemia and insulin resistance [37]. Also, an increase in glycemic load occurred simultaneously with exacerbated levels of inflammatory biomarkers such as interleukin-6, linked to insulin resistance [38]. Furthermore, the effects of fructose as the main constituent of additive nutritive sweeteners such as high fructose corn syrup and sucrose on metabolic profile are well defined in animal and human studies [39, 40]. High fructose diet increased lipogenesis, leptin resistance, intra-abdominal fat storage, hunger rating, energy intake, CRP concentration, blood pressure, and induced impaired fasting glucose by insulin resistance in the liver and adipose tissue [40, 41]. Fructose is preferentially metabolized to lipid in the liver leading to increased triglyceride levels, which have been associated with development of insulin resistance [42]. Moreover, fructose increased serum uric acid and enhanced reabsorption of salt and water in the small intestine and kidney, indicating the hypertension-induced effects of fructose [43].

Our study has both strengths and limitations. Some limitations of our analysis deserve comment; using an FFQ cannot estimate the ‘actual’ intake of participants. However, we used the FFQ to rank individuals based on their habitual intakes of SSBs. Also, the low-resolution capacity of FFQ that relays in memory of food consumption is critical at any age but, worst in children. However, for the children and adolescents, FFQ is an instrument, which can be administered in large populations and be accurate, reproducible, and relatively inexpensive for repeated measures. Several studies show that past dietary intake of children and adolescents can be measured reasonably well by FFQ [44–46]. Using well-trained interviewers who are skilled in the art of asking questions that help subjects remember what they ate and asking mothers about the type and quantity of meals and snacks when children were unable to recall may help minimizing the memory limitations. Another limitation was the lack of data on puberty stage. In addition, the fruit juice drink item in the FFQ was not assessed separately for 100 % fruit juice and sugar sweetened synthetic juice drinks that are not 100 % juice. Furthermore, the SSSD item assessed in the FFQ did not differentiate between the artificial non-caloric sweeteners and those containing caloric sugar e.g. fructose or sucrose. As for strengths, despite the limitations, to our knowledge, this is the first study to evaluate SSB consumption in relation to incidence of MetS after a 3.6 year follow-up among children and adolescents. Longitudinal design of the study allowed us to confirm the role of SSB consumption on the incidence of MetS. Furthermore, collection of socio-demographic information of the population-based cohort subjects facilitated a thorough investigation of possible confounders.

## Conclusion

Our results examining the prospective associations provide evidence that greater SSB consumption during childhood and adolescence may have undesirable effects on cardio-metabolic risk factors, some of which may be independent of BMI. The results suggest that SSB particularly SSSD consumption should be limited during early years to prevent future occurrence of MetS. Although SSBs are a disposable component of world’s modern diet, findings of the current study and those from systematic reviews on the contribution of SSBs on health provide strong evidence of harm. We should not wait for absolute proof and healthy alternatives to SSBs should be promoted.

## Abbreviations

MetS: Metabolic syndrome
SSB: Sugar sweetened beverage
TLGS: Tehran Lipid and Glucose Study
TG: Triglyceride
HDL-C: High density lipoprotein cholesterol
FPG: Fasting plasma glucose
FFQ: Food frequency questionnaire
USDA: United States department of agriculture
SSSD: Ssugar sweetened carbonated soft drink
DR: Dietary recall
MAQ: Modifiable activity questionnaire
MET: Metabolic equivalent task
BMI: Body mass index
WC: Waist circumference
OR: Odds ratio
CI: Confidence interval
